# Recombinant Human Activated Protein C in the Treatment of Acute Respiratory Distress Syndrome: A Randomized Clinical Trial

**DOI:** 10.1371/journal.pone.0090983

**Published:** 2014-03-14

**Authors:** Alexander D. Cornet, A. B. Johan Groeneveld, Jorrit J. Hofstra, Alexander P. Vlaar, Pieter R. Tuinman, Arthur van Lingen, Marcel Levi, Armand R. J. Girbes, Marcus J. Schultz, Albertus Beishuizen

**Affiliations:** 1 Department of Intensive Care, VU University Medical Center, Amsterdam, The Netherlands; 2 Department of Internal Medicine, VU University Medical Center, Amsterdam, The Netherlands; 3 Institute for Cardiovascular Research ICaR-VU, VU University Medical Center, Amsterdam, The Netherlands; 4 Department of Intensive Care, Erasmus Medical Center, Rotterdam, The Netherlands; 5 Department of Intensive Care, Academic Medical Center, Amsterdam, The Netherlands; 6 Laboratory of Experimental Intensive Care and Anesthesiology, Academic Medical Center, Amsterdam, The Netherlands; 7 Department of Internal Medicine, Academic Medical Center, Amsterdam, The Netherlands; 8 Department of Nuclear Medicine, VU University Medical Center, Amsterdam, The Netherlands; 9 Department of Intensive Care, Medisch Spectrum Twente, Enschede, The Netherlands; University Hospital Basel, Switzerland

## Abstract

**Rationale:**

Pulmonary coagulopathy may play a pathogenetic role in acute respiratory distress syndrome (ARDS), by contributing to alveolocapillary inflammation and increased permeability. Recombinant human activated protein C (rh-APC) may inhibit this process and thereby improve patient outcome.

**Methods:**

A prospective randomized, saline-controlled, single-blinded clinical trial was performed in the intensive care units of two university hospitals, and patients with ARDS were included within 24 h after meeting inclusion criteria.

**Intervention:**

A 4-day course of intravenous rh-APC (24 mcg/kg/h) (n = 33) versus saline (n = 38).

**Outcomes:**

The primary outcome parameter was the pulmonary leak index (PLI) of ^67^Gallium-transferrin as a measure of alveolocapillary permeability and secondary outcomes were disease severity scores and ventilator-free days, among others.

**Results:**

Baseline characteristics were similar; in 87% of patients the PLI was above normal and in 90% mechanical or non-invasive ventilation was instituted at a median lung injury score of 2.5. There was no evidence that Rh-APC treatment affected the PLI or attenuated lung injury and sequential organ failure assessment scores. Mean ventilator-free days amounted to 14 (rh-APC) and 12 days (saline, P = 0.35). 28-day mortality was 6% in rh-APC- and 18% in saline-treated patients (P = 0.12). There was no difference in bleeding events. The study was prematurely discontinued because rh-APC was withdrawn from the market.

**Conclusion:**

There is no evidence that treatment with intravenous rh-APC during 4 days for infectious or inflammatory ARDS ameliorates increased alveolocapillary permeability or the clinical course of ARDS patients. We cannot exclude underpowering.

**Trial Registration:**

Nederlands Trial Register ISRCTN 52566874

## Introduction

Acute respiratory distress syndrome (ARDS), with its milder form formerly known as acute lung injury (ALI), occurs in 30 to 80 per 100,000 person-years and is a major cause of morbidity and mortality in the critically ill [1], [2]. Treatment of ARDS is supportive since there are no routine drugs for treatment, other than treatment of the underlying disease [3]. A key factor in the pathogenesis of ARDS is alveolocapillary inflammation, leading to endothelial barrier dysfunction and increased permeability, that can be assessed at the bedside, with help of the non-invasively measured pulmonary leak index (PLI) of ^67^Gallium (^67^Ga)-transferrin [4]–[6]. In previous studies it was demonstrated that the PLI parallels the clinical severity and course of ARDS, for instance expressed as changes in the lung injury score [5]. Furthermore, the PLI appeared to be more accurate in assessing the degree of permeability than extravascular lung water measurements [7].

There is an extensive crosstalk between inflammation, activated coagulation and depressed fibrinolysis, so that alveolar fibrin depositions and small vessel thrombi are thought to contribute and perpetuate alveolocapillary inflammation, pulmonary vascular injury and barrier dysfunction [3], [8]–[11]. The alveolar and systemic levels of naturally occurring anticoagulants, such as activated protein C (APC), may be depressed because of consumption, impaired synthesis and degradation, and inhibitors of fibrinolysis may be increased, and both phenomena may be associated with pulmonary and remote organ dysfunction and mortality [8], [9]. In healthy volunteers, infusion of rh–APC attenuated coagulopathy and neutrophils in the lungs after inhalation of endotoxin [12], [13]. This is in line with beneficial effects of rh–APC infusion in models of sepsis and ARDS on pulmonary coagulopathy and consequently on alveolocapillary inflammation, as well as with directly ameliorating effects on endothelial barrier dysfunction via stimulation of protease-activated receptor-1 (PAR-1), protein C and sphingosine-1-phosphate (S1P) receptors in the endothelium [11], [14], [15]. The latter may downregulate, among others, pulmonary endothelial release of angiopoietin-2 that may play a direct role in the increased permeability in patients with ARDS, and may attenuate cytoskeletal rearrangement via Rho-associated kinase [11], [14]–[17]. In patients with severe sepsis, often accompanied by ARDS, infusion of recombinant human (rh) APC reduced mortality by ameliorating organ dysfunction, including respiratory dysfunction as demonstrated in two multicenter trials (PROWESS, ENHANCE) [18], [19], [20]. Of note, infusion was particularly effective in patients who presented with lung infection, community–acquired pneumonia or need for mechanical ventilation [21], [22]. In a recent large study in patients with septic shock (PROWESS SHOCK), rh-APC appeared of no benefit and was withdrawn from the market after publication, although two prior multicenter trials (ADDRESS, RESOLVE) already raised concerns regarding its efficacy [23]–[25]. About 43% had a pulmonary origin of sepsis in the PROWESS-SHOCK trial. In a recent meta-analysis, including the aforementioned negative trial [25], however, the drug was suggested to maintain effectiveness [26].

For the current study, performed before publication of the last multicenter study on APC [25], we hypothesized that infusion of rh–APC attenuates the increase in pulmonary vascular permeability and thereby benefits patients with ARDS as a single organ failure. We performed a single-blinded, randomized controlled multicenter trial of patients with ARDS comparing intravenous infusion of rh–APC with saline, studying the effect on the PLI as primary outcome measure [4]–[6]. Secondary outcomes included lung injury score (LIS) and sequential organ failures score (SOFA), duration on mechanical ventilation and ventilator-free days, and mortality. A substudy of our trial was recently published and suggested attenuated hypercoagulability, increased fibrinolysis and thereby less lung injury by rh-APC treatment [27].

## Patients and Methods

### Study design

This is a report of the infectious and inflammatory ALI/ARDS (INFALI) trial, a multicenter prospective, single-blinded, randomized, saline-controlled clinical trial in patients with ALI/ARDS (trial registration number ISRCTN 52566874). The patients were blinded for the allocated treatment. The Ethics Committee of the VU University Medical Center, Amsterdam, the Netherlands, approved the study protocol. Written informed consent was obtained from all patients or their next of kin before enrolment in the trial. All clinical investigations have been conducted according to the principles expressed in the Declaration of Helsinki. The protocol for this trial and supporting CONSORT checklist are available as supporting information; see Checklist S1 and Protocol S1.

### Inclusion and exclusion criteria

Patients, over 18 years of age and admitted to the mixed medical–surgical intensive care units (ICU's) of two participating university medical centers in Amsterdam, were to be included because of respiratory insufficiency within 24 hours after diagnosis of ALI/ARDS, of any cause, including pneumonia, sepsis, aspiration according to standard clinical criteria, irrespective of the need for ventilatory support. The definition used to establish the diagnosis pneumonia was radiographic evidence of pulmonary consolidation in association with the production of purulent sputum with plus two positive SIRS criteria (1. core temperature of ≥38°C or ≤36°C; 2. heart rate of ≥90 beats/min; 3. respiratory rate ≥20 breaths/min or a PaCO_2_ ≤32 mmHg or the use of mechanical ventilation for an acute respiratory process; 4. white cell count ≥12,000/mm^3^ or ≤4,000/mm^3^ or a differential count showing >10% immature neutrophils) [28]. This was adjudicated by ADC, JJH, MJS and AB. ALI/ARDS was diagnosed using the North American European Consensus Conference (NAECC) definition [29]. Although inclusion was on the basis of ALI/ARDS, we recoded conditions according to the current Berlin definition of ARDS, according to variables at enrollment [2]. Patients were excluded if rh–APC treatment was indicated based on national guidelines at the time of the study (i.e., severe sepsis or septic shock, acute physiology, age and chronic health evaluation II score (APACHE II) score ≥25 and in the absence of informed consent [30]. Additional exclusion criteria were: platelet count <30×10^9^/L, any major surgery within 12 hours before inclusion, acute bleeding, severe head trauma, intracranial surgery or stroke within 3 months before inclusion, known intracranial abnormalities (e.g., malignancies or other tumors, arteriovenous malformation), known hypercoagulability (e.g., protein C resistance, hereditary deficiency of protein C, protein S or antithrombin, or anticardiolipin– or antiphospholipid–antibodies), congenital hemorrhagic diathesis, pregnancy or breast feeding, liver cirrhosis with portal hypertension and/or esophageal varices, presence of an epidural catheter; severely immune–compromised status (e.g., HIV–infected patients with CD4 count <50/mL, and patients treated with immunosuppressive medication following bone marrow or solid organ transplantation). The following concomitant medications were reasons for exclusion: heparin in therapeutic dose (within 8 hours of study entry), coumarin derivatives at any dose (within 7 days of study entry), acetylsalicylic acid at a dose >650 mg/day (within previous 3 days of study entry), thrombolytic therapy at any dose (within previous 3 days of study entry), glycoprotein IIb/IIIa inhibitors at any dose (within 7 days of study entry), antithrombin at any dose (within 3 days of study entry) and previous treatment with rh–APC (at any time within study entry). Prophylactic dose of low molecular weight heparin was allowed.

### Treatment protocol

All patients were treated by the discretion of the supervising intensivists according to international guidelines. If needed, mechanical ventilation was performed after endotracheal intubation, in a pressure–controlled mode, aiming at a maximum airway pressures <35 cmH_2_O, and tidal volumes ≤6 mL/kg predicted ideal body weight (Devine formula), with or without proning, when indicated on clinical grounds. Patients receiving mechanical ventilation after endotracheal intubation underwent selective decontamination of the digestive tract after collection of tracheal aspirate cultures, oropharyngeal and perineal swabs. Antibiotic therapy was guided by Gram–stains and cultures, according to local guidelines for antimicrobial therapy. Fluid therapy consisted of crystalloids, with or without gelatins and/or hydroxyethyl starches, in order to maintain arterial blood pressure (MAP >70 mmHg) and diuresis (>30 mL/h).

### Study protocol

Patients were randomly assigned to infusion of rh–APC or a similar volume of normal saline. Prior to the start of the trial sealed opaque envelopes, containing the treatment assignment for each patient, were numbered through block randomization, with 6 blocks of patients, stratified per participating unit. Open label rh–APC (Eli Lilly, Indianapolis, IN, USA), at a dose of 24 mcg/kg/h, or saline was infused at a constant rate for a total of 96 hours, starting within 6 hours after randomization. Randomization was within 12 h after meeting above inclusion criteria. Infusion of rh–APC was interrupted 1 hour before any invasive percutaneous procedure or major surgery. When no bleeding complications occurred, infusion of rh–APC was resumed 1 hour after a percutaneous procedure, and 12 hours after major surgery, in line with international guidelines. All patients completed the 96-hour treatment. No patient met the criteria for APC administration according to the national guidelines prevailing at the time of the study.

### PLI

The PLI was measured within 0–4 hours prior to the start of infusion of the study drug or saline, and repeated within 12 hours following the end of 96 hour infusion, according to published methods [4]–[6]. Transferrin was labeled in vivo, after intravenous injection of 4–5 MBq ^67^Ga-citrate (physical half-life 78 hrs; Mallinckrodt Diagnostica, Petten, the Netherlands). Patients were in supine or prone position, and two scintillation probes (Eurorad C.T.T., Strasbourg, France) were placed over the left and right lung apices. Starting from the time of ^67^Ga injection, radioactivity was measured for 30 minutes. The ^67^Ga counts are corrected for background activity, physical half-life of ^67^Ga and decay after injection, and expressed as counts per minute per lung. At 0, 5, 8, 12, 15, 20, 25 and 30 minutes, blood samples were taken. Each blood sample was weighed and radioactivity was measured with a single-well well-counter (LKB Wallac 1480 WIZARD, Perkin Elmer, Life Science, Zaventem, Belgium) taking background and physical half-life into account. Results are expressed as counts per minute per gram. For each blood sample, a time-matched counts per minute over each lung was taken. The radioactivity ratio was calculated as (^67^Ga_lung_)/(^67^Ga_blood_) and plotted against time. The PLI was calculated from the slope of the increase of the radioactivity ratio, divided by the intercept, to correct for physical factors in radioactivity detection and pulmonary blood volume. The PLI thus represents the transport rate of ^67^Ga-transferrin from the intravascular to the extravascular space of the lungs and is therefore a measure of pulmonary vascular permeability. The values for both lungs were averaged. The upper limit of normal for the PLI is 14.1×10^−3^/min, and the measurement error (coeffecient of variation if measurement is repeated in the same patient) is approximately 10% [31].

### Data collection

Upon enrolment, data on baseline demographics, comorbidity and reasons of admission to the intensive care unit (ICU), as well as hemodynamic and respiratory parameters were collected. The APACHE II [30], the simplified acute physiology score (SAPS II)[32], the sequential organ failure assessment score (SOFA) [33] and the lung injury score (LIS) [34] were calculated from worst values in the 24 h preceding enrolment and, for SOFA and LIS, on day 5 and 15 after enrolment. For the LIS we evaluated daily chest radiographs and scored the number of consolidated quadrants. From the blood gas measurements, done for routine care, daily worst values were taken and also the worst ventilatory settings were taken from the patient data management system available in the units. Total respiratory dynamic compliance was calculated from tidal volume/(peak inspiratory pressure - positive end expiratory pressure), mL/cm H_2_O. We estimated in patients not on mechanical ventilation the inspiratory O_2_ fraction (F_I_O_2_) from liters of O_2_ administered nasally or via non-rebreathing mask, varying between 1 and 15 L, yielding an estimated F_I_O_2_ from 23 to 70%, respectively. The number of ventilator-free days (VFD) was defined as the number of days with unassisted breathing (>24 h) from randomization to day 28 after enrolment. Patients who died before day 28 while receiving ventilator support, were assigned zero ventilator-free days [35]. Lengths of stay and mortality at day 28 and 90 were recorded, within or outside the ICU or the hospital.

### Statistical analysis

The study was powered (at 80%) to include 96 patients to detect an anticipated difference in PLI of 20% at a standard deviation (SD) of 40% (α = 0.05). The Kolmogorov-Smirnov test was used to check for normal data distribution (if P>0.05). Data were expressed as means (± standard deviation) for normally distributed data, medians (± interquartile range) for non-normally distributed data, or absolute numbers where appropriate. Nonparametric data were analyzed using Mann–Whitney U and categorical data by Fisher's exact test. The Spearman rank correlation was used to express relations. Kaplan-Meier plots were made and a log rank test performed for ventilatory independency and survival in time in the groups. A Cox proportional-hazards model was used to estimate the hazard ratio (HR) for death with the use of rh-APC versus saline in different posthoc defined subgroups (with 95% confidence intervals). A P value of <0.05 was considered statistically significant and exact values are given unless <0.001. Statistical analysis was performed using SPSS 19.0 (SPSS, Chicago, IL, USA) and Prism 5.0 (GraphPad Software, San Diego, CA, USA).

## Results

**Figure 1:**
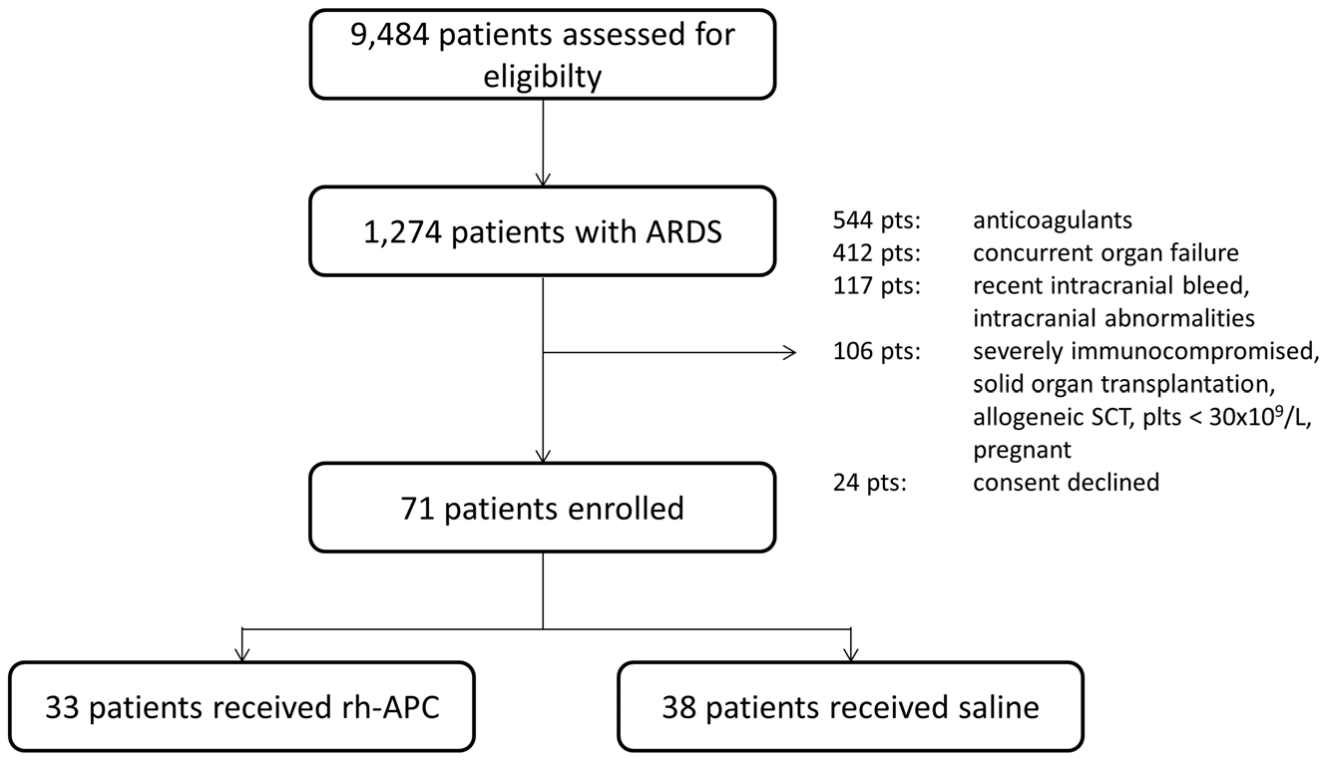
CONSORT diagram. ARDS, acute respiratory distress syndrome; SCT, stem cell transplantation; plts, platelet count.

Between 1 January 2007 and 1 May 2011 9,484 patients were assessed for eligibility (Fig. 1). Of these patients, 71 patients were enrolled in the study. Reasons for exclusion are given in Fig. 1. There were 33 patients assigned to rh-APC and 38 to saline. The study was prematurely discontinued because rh-APC was withdrawn form the market and no longer commercially available.

### Baseline characteristics

Patient groups did not differ with regard to demographic and baseline parameters (Table 1). In 61 patients the reason for inclusion was pneumonia. There was a trend towards more pulmonary comorbidity in the saline-treated group. With regard to disease severity as expressed by APACHE II and SAPS II scores, groups did not differ. Furthermore, the frequency of treatment with vasopressors and steroids was similar. In the majority of patients (56/71) tracheal aspirate cultures were positive. In 14 patients (n = 5 rh-APC and n = 9 saline) multiple pathogens were isolated. *Streptococcus pneumoniae* was the most prevalent identified micro-organism, both in tracheal aspirate and in blood cultures. Ninety percent of the patients (64/71) needed invasive mechanical ventilation.

**Table 1:** Baseline characteristics.

	rh-APC (n = 33)	saline (n = 38)	P-value
Age	62.2±14.4	60.6±17.7	0.940
Sex (male)	15 (45)	25 (66)	0.099
Height (cm)	168.5±8.7	172.8±9.6	0.097
Weight (kg)	73.9±14.9	71.6±16.3	0.518
Comorbidities			
Cardiovascular	15 (45)	21 (55)	0.479
Pulmonary	5 (15)	14 (37)	0.059
Renal	1 (3)	3 (8)	0.618
malignancy	4 (12)	6 (16)	0.739
Etiology of ARDS			
pneumonia	27 (82)	34 (89)	0.242
abdominal sepsis	3 (9)	1 (3)	
near-drowning	0	2 (5)	
smoke inhalation	2 (6)	0	
miscellaneous	1 (3)	1 (3)	
Severity of ARDS (Berlin criteria)			
mild	9 (27)	12 (32)	0.582
moderate	23 (70)	23 (61)	
severe	1 (3)	3 (7)	
Blood stream infection	6	10	0.459
* Streptococcus pneumoniae*	3	4	
* Listeria monocytogenes*	1	0	
* Enterococcus faecium*	0	1	
Coagulase-negative staphylococcus	2	5	
Tracheal aspirate			
* Streptococcus pneumoniae*	3	8	0.685
* Beta-haemolytical streptococcus*	1	-	
* Staphylococcus aureus*	2	6	
* Enterococcus faecalis*	1	-	
* Listeria monocytogenes*	1	-	
* Escherichia coli*	3	4	
* Pseudomonas aeruginosa*	4	1	
* Proteus mirabilis*	-	2	
* Hafnia alveii*	2	-	
* Enterobacter cloacae*	1	-	
* Haemophilus influenzae*	1	2	
* Klebsiella oxytoca*	1	-	
* Klebsiella pneumoniae*	-	3	
* Aeromonas spp*	-	1	
* Pneumocystis jirovecii*	1	-	
* Aspergillus spp*	2	1	
* Candida spp*	2	3	
Disease severity			
APACHE II	17.3±6.2	16.9±5.4	0.707
SAPS II	41.5±12.8	37.8±12.8	0.266
SOFA	7.3±2.3	7.0±2.0	0.867
Vital signs			
Temperature (°C)	36.6±1.4	36.5±1.7	0.703
Heart rate (/min)	101.0±32.2	112.3±27.8	0.121
MAP (mmHg)	69.9±11.7	69.0±16.2	0.503
Treatment			
Vasopressors	26 (79)	30 (79)	1.000
Corticosteroids	23 (70)	24 (63)	0.800
Duration between admission and start of study (days)	1.8±3.4	1.7±2.4	0.595

Mean/median ± standard deviation/interquartile range, respectively, or number (percentage), where appropriate. Rh-APC, recombinant human activated protein C; APACHE, acute physiology and chronic health evaluation; SAPS, simplified acute physiology score; SOFA, sequential organ failure assessment; MAP, mean arterial pressure.

### Pulmonary variables

At baseline, the PLI was increased as compared to normal values in 87% (62/71) of patients. The baseline PLI and LIS, which did not differ among groups, correlated at R_s_ = 0.26, P = 0.030 (Table 2). The baseline LIS was associated with the duration of mechanical ventilation (R_s_ = 0.33, P = 0.005). There were no differences between groups in the course of ventilator pressures, tidal volumes, gas exchange, and oxygen requirements.

**Table 2:** Pulmonary variables.

	Baseline)			Day 5			Day 15		
	rh-APC	saline	P	rh-APC	saline	P	rh-APC	saline	P
	(n = 33)	(n = 38)		(n = 33)	(n = 38)		(n = 19)	(n = 11)	
**Gasometrics**									
PaO_2_, mmHg	88.0±21.5	88.9±23.0	0.921	88.8±19.2	90.7±27.7	0.756	89.7±17.8	84.8±7.7	0.713
F_I_O_2_, %	53.1±12.5	55.2±16.8	0.924	43.9±9.9	44.3±11.2	0.965	41.4±10.8	40.8±6.7	0.868
PaO_2_/F_I_O_2_	175.1±48.6	170.7±53.8	0.624	220.2±72	217.7±95.4	0.732	227.7±69.8	213.8±41.3	0.483
PaCO_2_, mmHg	45.7±12.5	47.2±11.6	0.604	43.3±9.7	45.6±9.3	0.393	45.2±11.91	44.4±5.3	0.867
pH	7.36±0.08	7.34±0.10	0.390	7.44±0.05	7.42±0.07	0.502	7.43±0.04	7.44±0.02	0.524
**Ventilation**									
Mode			0.551			0.551			0.702
Unassisted breathing	4 (12)	3 (8)		4 (12)	3 (8)		7 (37)	3 (27)	
Invasive ventilation	29 (88)	34 (89)		29 (88)	34 (89)		12 (63)	8 (73)	
Non-invasive ventilation	0	1 (3)		0	1 (3)		0	0	
Prone position	5 (15)	11 (29)	0.255	5 (15)	11 (29)	0.255	1 (5)	0	1.000
Respiratory rate (/min)	25.8±6.3	25.5±6.1	0.766	20.8±4.9	22.6±4.8	0.288	23.8±4.8	23.4±4.1	0.928
PIP (cm H_2_O)	32.0±9.3	31.6±7.5	0.989	26.0±8.8	26.2±8.4	0.825	23.3±10.5	18.4±6.1	0.384
PEEP (cm H_2_O)	12.3±4.6	12.8±4.3	0.819	11.0±4.3	10.7±4.2	0.839	9.8±3.8	8.5±2.8	0.482
Tidal volume (mL)	459±96	451±120	0.544	449±76	476±77	0.193	430±65	407±74	0.571
Tidal volume (mL/kg IBW)	7.5±1.8	6.8±2.0	0.091	7.4±1.4	7.3±1.9	0.628	7.4±1.1	6.3±1.9	0.135
Compliance (mL/cm H_2_O)	24.5±7.8	24.3±6.5	0.843	33.5±13.4	32.9±11.7	0.944	40.8±23.1	50.8±29.6	0.343
Chest radiograph quadrants	2.2±0.9	1.9±0.7	0.249	1.7±0.8	1.6±0.8	0.851	1.0±1.1	1.5±1.2	0.245
**Lung injury**									
PLI (x10^-3^/min)	33.8±20.7	31.2±20.6	0.335						
Lung injury score	2.5±0.7	2.5±0.6	0.862						
									

Mean or median ± standard deviation or interquartile range, respectively, or number (percentage), where appropriate. Rh-APC, recombinant human activated protein C; PaO_2_, partial pressure of O_2_; F_I_O_2_, inspiratory O_2_ fraction; PIP, peak inspiratory pressure; PEEP, positive end-expiratory pressure; IBW, ideal body weight; PLI, pulmonary leak index; LIS, lung injury score (between 0 and 4).

### Primary and secondary outcome measures

**Figure 2:**
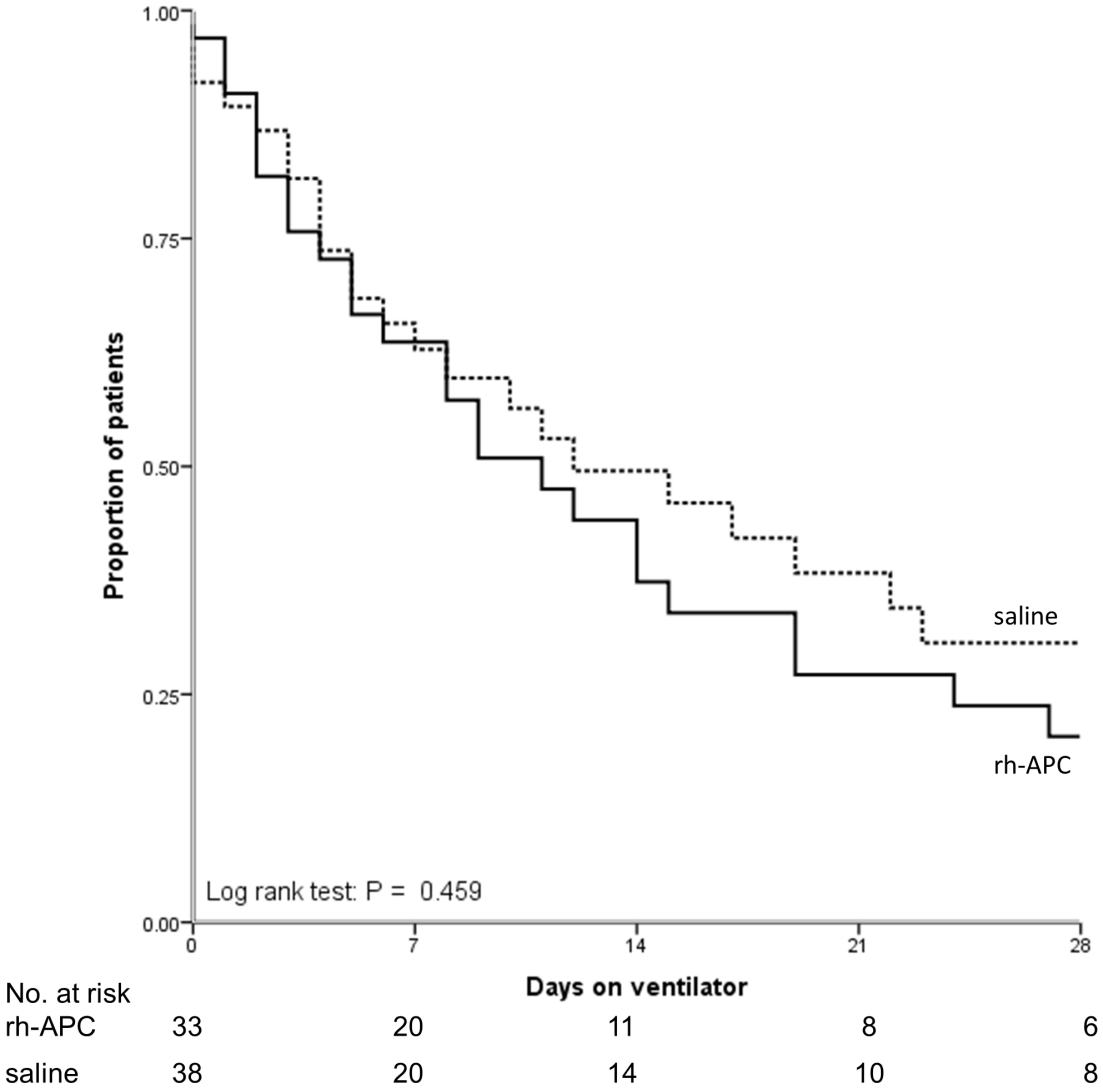
Probability of being alive and off the ventilator until day 28 in study groups.

**Figure 3:**
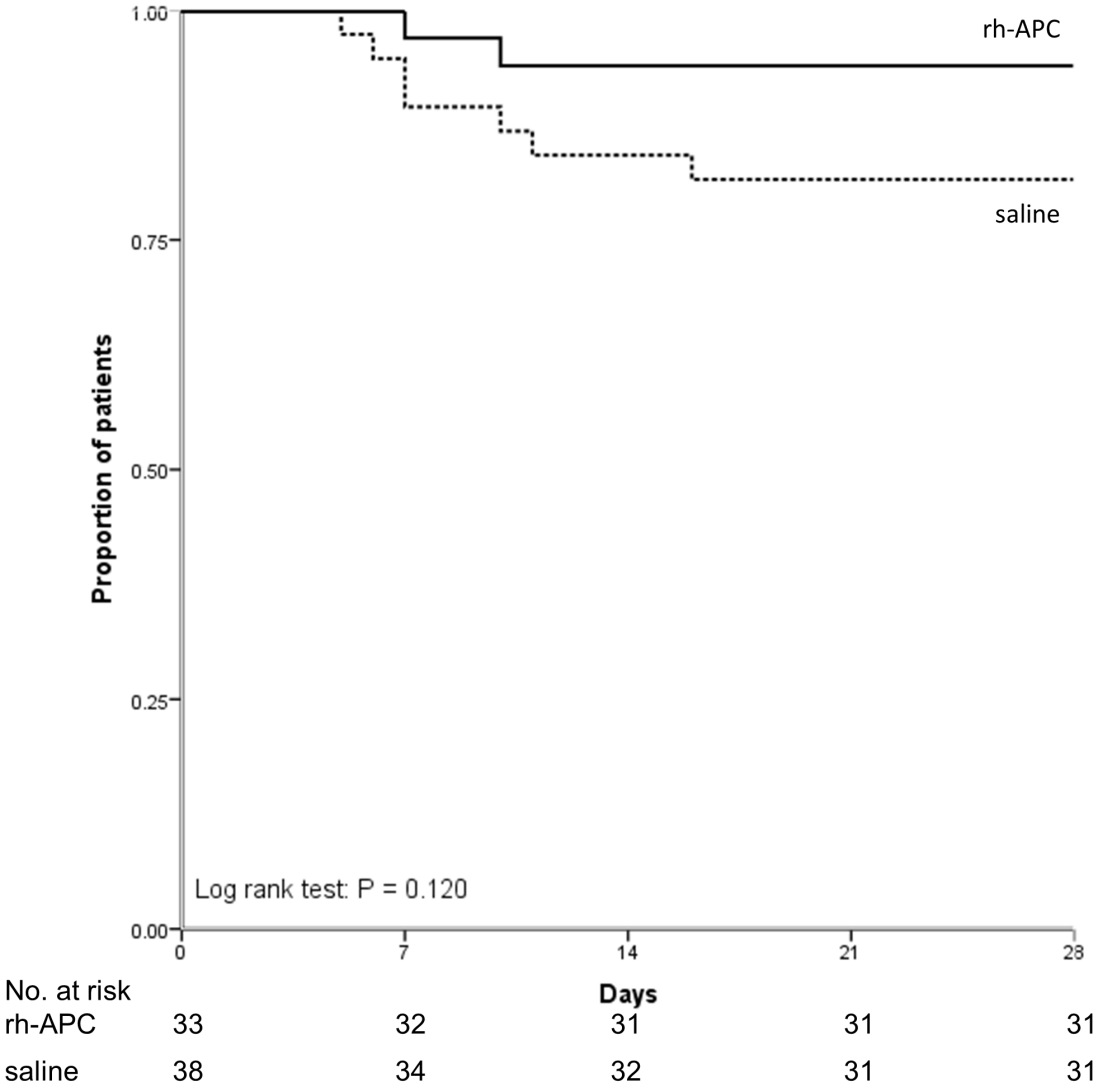
Probability of survival until day 28 in study groups.

**Figure 4:**
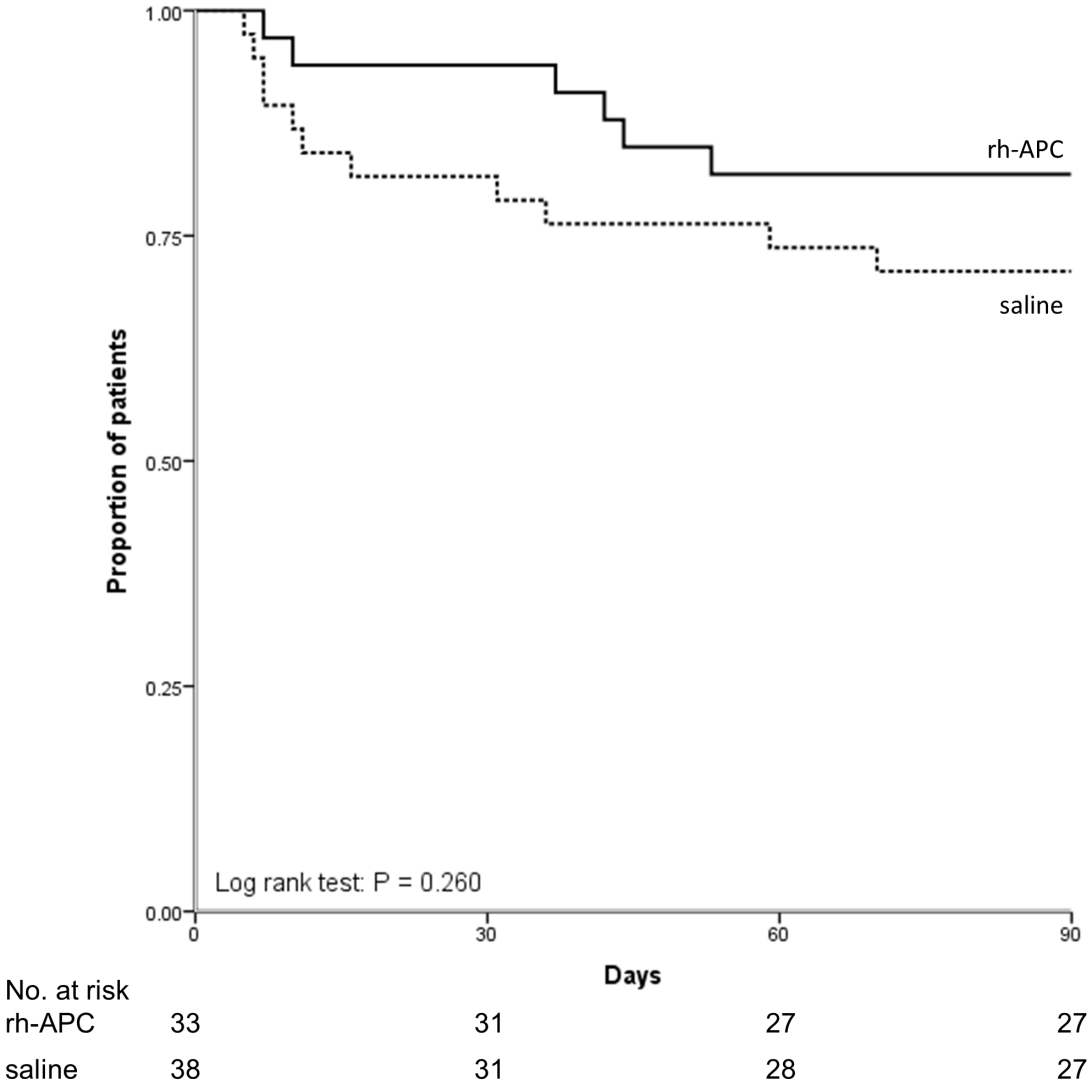
Probability of survival until day 90 in study groups.

Table 3 shows that there is no difference in day 5 PLI between treatment groups, although the reduction in PLI was more pronounced in the rh-APC group, yet not reaching statistical significance. There was no effect of rh-APC on the general disease severity score (SOFA) nor the more lung-specific LIS and the number of ventilator-free days. Fig. 2 shows the lack of difference in ventilator-dependency in the groups until day 28 after randomization. The day 5 LIS score was associated with the duration of mechanical ventilation (R_s_ = 0.58, P<0.001). With regard to mortality, no differences were found between treatment groups (Table 3 and Fig. 3 & 4).

**Table 3:** Primary and secondary outcome measures.

	rh-APC (n = 33)	saline (n = 38)	P-value
**Primary outcome**			
PLI day 5, ×10^−3^/min	26.2±16.0	27.0±15.8	0.878
Decrease PLI day 1–5, ×10^−3^/min	9.1±24.2	4.6±18.1	0.619
**Secondary outcomes**			
LIS day 5	1.8±0.9	1.9±1.0	0.654
LIS day 15	1.5±1.1	1.8±1.0	0.327
Ventilator-free days (days)	14.5±10.5	12.0±11.3	0.348
Duration of ventilation (days)	12.4±9.9	12.2±10.0	0.958
SOFA day 5	5.4±3.2	5.2±3.5	0.744
SOFA day 15	4.5±2.5	3.8±2.5	0.396
28-day mortality	2 (6)	7 (18)	0.157
ICU mortality	5 (15)	11 (29)	0.255
90-day mortality	6 (18)	11 (29)	0.404
Hospital mortality	7 (21)	12 (32)	0.423

Mean or median ± standard deviation or interquartile range, respectively, or number (percentage), where appropriate. Rh-APC, recombinant human activated protein C; PLI, pulmonary leak index; LIS, lung injury score; SOFA, sequential organ failure assessment; ICU, intensive care unit.

### Post-hoc subgroup analysis

Cox regression analysis did not identify any subgroup in which treatment with rh-APC resulted in a statistically significant survival benefit, even though all HR were below 1 (Table 4). In patients with pneumonia and supranormal PLI the P for 28-day survival with log-rank testing was 0.045 in favor of rh-APC.

**Table 4:** Hazard ratios for death on day 28.

	No. of	No. of deaths (%)		HR (95% CI)
	patients	rh-APC	saline	
All patients	71	2 (6)	7 (18)	0.310 (0.064–1.492)
Etiology of ARDS				
pneumonia	61	1(4)	7 (20)	0.165 (0.020–1.342)
other	10	1 (17)	0	-
Berlin criteria for ARDS				
mild	21	0	2 (17)	-
moderate/severe	50	2 (8)	5 (19)	0.414 (0.080–2.136)
Pulmonary leak index				
above normal	60	2 (7)	7 (22)	0.304 (0.063–1.462)
≥2× upper limit of normal	32	1 (6)	4 (27)	0.208 (0.023–1.861)
<2× upper limit of normal	39	1 (7)	3 (13)	0.511 (0.053–4.917)
≥2.5× upper limit of normal	22	0	4 (36)	-
<2.5× upper limit of normal	49	2 (10)	3 (11)	0.865 (0.145–5.176)
Lung injury score				
≥2.5	39	2 (11)	4 (19)	0.577 (0.106–3.152)
<2.5	32	0	3 (18)	-
Sequential organ failure assessment				
≥7	48	2 (9)	6 (24)	0.334 (0.067–1.658)
<7	23	0	1 (8)	-
Baseline steroids				
Yes	47	2 (9)	6 (25)	0.320 (0.064–1.583)
no	24	0	1 (7)	-
C-reactive protein				
≥175 mg/L	32	1 (63)	4 (25)	0.233 (0.026–2.089)
<175 mg/L	37	1 (7)	3 (14)	0.461 (0.048–4.429)

ARDS, acute respiratory distress syndrome; HR, hazard ratio with 95% confidence intervals.

### Adverse events

Two pneumothoraces occurred during the study, one in each patient group. There were no bleeding complications.

## Discussion

Our study suggests that a 4-day course of intravenous rh-APC does not ameliorate the increased permeability and clinical course of ARDS in critically ill patients. However, our study was underpowered.

The study was designed with the hypothesis that APC plays a role in the endothelial barrier function in the lung. The study was powered for a 20% change in PLI since increased alveolocapillary permeability was considered central in the pathogenesis and clinical presentation of ARDS [4]–[6]. We previously demonstrated that the PLI increases before ARDS becomes clinically manifest and declines when it resolves [4]. Our current study again documents that increased permeability is associated with the clinical manifestations of ARDS expressed as the LIS, as noted before [4]–[6], and that the latter is a determinant of duration of ventilatory support. Yet, in a substudy of this trial, we demonstrated that rh-APC infusion actually attenuates pulmonary coagulopathy [27]. Apparently, this effect on pulmonary coagulopathy does not result in a clinically significant enhancement of barrier function as expressed by the PLI. Therefore, we could not find evidence for the concept that rh-APC ameliorates endothelial barrier dysfunction and increased permeability and thereby attenuates the course of ARDS in man, as suggested by preclinical studies via a cytoprotective effect involving PAR-1 and S1P pathways, irrespective of anti-inflammatory effects [11], [14], [15]. In some animal studies (rats with pulmonary infection) intravenous administration of rh-APC limited bronchoalveolar coagulation, whereas it did not exert anti-inflammatory effects [36], [37].

The 28-day mortality rate of patients in our study was relatively low (13%), likely attributable to a lower overall disease severity, as severe sepsis, septic shock and APACHE II ≥25 were exclusion criteria, when compared with large international trials on ARDS that did not exclude the latter patients and reported mortality rates of 25 to 46% [38], [39]. It was however comparable to the 60-day mortality rate of 13% in the trial of Liu et al., who applied similar inclusion criteria for the 75 patients in their study, of whom only 40% had pneumonia [35]. Additionally, our study is in line with the results from the ADDRESS (Administration of Drotrecogin Alfa (Activated) in Early Stage Severe Sepsis) trial, focusing on patients with relatively low disease severity (APACHE II <25 or single organ failure) suffering from severe sepsis in whom rh-APC administration did not show clinical benefits [23]. In the double-blind, phase III, RESOLVE (REsearching severe Sepsis and Organ dysfunction in children; a gLobal perspectiVE) trial, 477 children with severe sepsis were enrolled. Again, there was no difference between rh-APC and placebo with regard to the composite time to complete organ failure resolution score. Mortality at 28 days was 17.2% in the rh-APC group versus 17.5% in the placebo group [24].

The limitations of our study include its premature discontinuation because rh-APC was withdrawn from the market, as described before [25]. The stringent exclusion criteria that we applied in order to reduce bleeding risks, contributed to the small number of patients that were enrolled. As a result, our study is underpowered to demonstrate amelioration of increased permeability and clinical course of ARDS in critically ill patients by intravenous rh-APC, as well as to demonstrate an effect on mortality, particularly in pneumonia-induced ARDS with increased alveolocapillary permeability. The single prior study on human ALI (n = 75), which also proved negative [35], was underpowered as well. Their case mix was more heterogeneous than in our study (only 40% had pneumonia) [35], suggesting that, when even in a more homogeneous population a benefit cannot be demonstrated, the contributory role of APC in ARDS must indeed be low. Nevertheless, our post hoc analyses, which should be interpreted with caution, serve to suggest the validity of trial design. The tidal volumes delivered to the patients in our study were larger than the 6 mL/kg ideal body weight described in the treatment protocol. However, the tidal volumes were comparable in both treatment groups throughout the study period. Moreover, the mean tidal volumes were within the range of 6 to 8 mL/kg ideal body weight, which is in keeping with the suggested lung-protective mechanical ventilation strategies in the Surviving Sepsis Campaing Guidelines [40].

The external validity of our study is compromised, as it was performed in 2 centers. The possibility of recruiting more centers was deemed impossible, both practically and logistically. The PLI measurements require highly specialized, custom-made scintillation probes. Furthermore, since many hospitals do not have a department of nuclear medicine, the isotopes would have needed to be transported via public roads for which government permission would have been needed, as well as additional permission for transportation through the hospital and administration in the ICU. Then the radioactive blood samples would have been needed to be transported back to one of both academic centers to perform the radioactivity count.

Our study is a single-blinded study. As a part of standard care, APTT and PT are regularly monitored in both centers. As rh-APC prolongs APTT, a truly double-blinded study was not considered feasible.

## Conclusion

In conclusion, this study suggests that treatment for 4 days with intravenous rh-APC for infectious or inflammatory ARDS does not ameliorate increased alveolocapillary permeability nor the clinical course of critically ill patients with ARDS as a single organ failure mostly caused by pneumonia.

### Key messages

• Increased pulmonary vascular permeability is associated with the clinical manifestations of ARDS
• Intravenous rh-APC for 4 days does not ameliorate increased alveolocapillary permeability nor the clinical course of critically ill patients with infectious or inflammatory ARDS as a single organ failure

## Supporting Information

Protocol S1 (DOC)

Checklist S1 (DOC)
