# Supplementary material for: Recombinant Human Activated Protein C in the Treatment of Acute Respiratory Distress Syndrome: A Randomized Clinical Trial
Source: PLoS One. 2014 Mar 14;9(3):e90983. doi: 10.1371/journal.pone.0090983 (PMC3954619; doi:10.1371/journal.pone.0090983)
Supplement: Protocol S1 — (DOC) [file pone.0090983.s001.doc]

## The INFALI study

**Activated Protein C versus placebo in the treatment of Inflammatory or Infectious ALI/ARDS (INFALI): a pathophysiological study on pulmonary microvascular permeability, apoptosis, inflammation and coagulation.**

*Principal Investigators:*

Albertus Beishuizen^1^, Marcus J. Schultz^2^, AB Johan Groeneveld^1^

Alexander D. Cornet^1^, Jan-Jorrit Hofstra^2^

*Co-investigators:*

Marijke Van Ham^3^, Marcel Levi^2^, Istvan Vermes^4^, Pieter GHM Raijmakers^5^, Arthur van Lingen^5^, Michiel A. van Agtmael ^6^, Armand RJ Girbes^1^

^1^ Department of Intensive Care, VUMC, Amsterdam

^2^ Department of Intensive Care, Academic Medical Center, Amsterdam

^3^ Department of Immunopathology, Sanquin Research, Amsterdam

^4^ Department of Clinical Chemistry, Medical Spectrum Twente, Enschede

^5^ Department of Nuclear Medicine, VUMC, Amsterdam

^6^Department of Internal Medicine and Infectious Disease, VUMC, Amsterdam

*Corresponding Investigator*:

A. Beishuizen, MD PhD

Department of Intensive Care

VU University Medical Center

PO Box 7057

1007 MB Amsterdam

Netherlands

Tel: 020-4442342

Fax: 020-4442392

e-mail: [beishuizen@vumc.nl](mailto:beishuizen@vumc.nl)

**Project overview/summary**

Activated protein C (aPC), an endogenous plasma serine protease with antithrombotic, profibrinolytic and anti-inflammatory properties is an important modulator of the host response to infection. APC was shown to reduce 28-day all cause mortality in patients with severe sepsis and to have an acceptable safety profile. The uncontrolled coagulation combined with uncontrolled systemic inflammation, which may lead to multiple organ dysfunction and lethal septic shock, are the primary targets of intervention with aPC.

In acute lung injury and the adult respiratory distress syndrome (ALI and ARDS, respectively) several features similar to sepsis are found such as microvascular thrombosis and a disrupted protein C system, both determinants of outcome in these patients. Considering these circulatory and intra-alveolar derangements which clearly contribute to the pathogenesis of ALI/ARDS (increased pulmonary dead space, decreased pulmonary blood flow) the protein C pathway could be a therapeutic target in ALI/ARDS. Because aPC is known to have anti-coagulant and anti-inflammatory properties it is plausible that aPC may have value in the treatment of patients with ALI from infectious and non-infectious origin.

Therefore, the **primary** aim of our study is to investigate the effect of aPC versus placebo on pulmonary microvascular permeability, extra-vascular lung water, gas exchange, the severity of lung injury, mode of ventilation and radiographic abnormalities in patients with ALI/ARDS by either infectious causes (unless the PROWESS/NVIC criteria are met *casu quo* only septic patients with single organ(respiratory) failure) or by inflammatory causes (pulmonary contusion, toxic, pancreatitis, vasculitis, etc). We will primarily stratify patients according to their etiology (infectious versus inflammatory) and secondly to the mode of ventilatory support (mechanical ventilation, noninvasive ventilation, spontaneous breathing). In addition, we will assess inflammatory, coagulation and fibrinolysis markers in both the systemic circulation as well as the alveolar compartment (when mechanically ventilated).

**Introduction**

Activated protein C (aPC) is an natural anticoagulant with anti-inflammatory properties. A recent placebo-controlled randomized trial has shown an impressive mortality benefit of aPC during severe sepsis and shock [1]. However, the exact mode of action of aPC is still unclear [2-5], both in terms of mechanisms (anticoagulation versus anti-inflammation) as well as effect on organ function mediating the observed survival benefit.

Acute lung injury (ALI) and acute respiratory distress syndrome (ARDS) are conditions similar to sepsis and have several common features with sepsis, including the frequent organ system dysfunction and apparent hypercoagulability with widespread microvascular thrombus formation [6,7]. In addition to microvascular thrombosis, there is also intra-alveolar activation of the coagulation cascade with deposition of fibrin along the injured alveolar surface (hyaline membranes). In these potentially lethal conditions, for which only supportive care is available, such as mechanical ventilation in the intensive care unit, this local imbalance of coagulation to fibrinolysis plays a significant role, by interacting with the proinflammatory changes resulting in the capillary-alveolar damage, which is characteristic for both ALI and ARDS [6-9]. The protein C system is, similar to sepsis, markedly affected in patients with ALI/ARDS from both septic and non-septic causes, as demonstrated by lower plasma/alveolar protein C levels and higher levels of thrombomodulin in pulmonary edema fluid [10].

The protein C pathway is central to outcome from severe sepsis and, likely, from ALI/ARDS [10,11]. The protein C pathway is known to be one of the main inhibitors of coagulation activation and subsequent thrombin generation: under normal conditions, activation of the protein C system may serve to prevent these deleterious effects of thrombin in the airways. The airway epithelial cell is a extravascular source of PC and provides a suitable microenvironment for efficient PC activation [12]. Several studies support the role of aPC in the regulation of inflammation by blocking the secretion of inflammatory cytokines from mononuclear cells and inhibition of leucocyte infiltration at sites of infiltration [11]. In an *in vivo* human model of endotoxin-induced acute pulmonary inflammation, aPC reduces the accumulation of neutrophils in the airspaces as well as chemotaxis [13]. The mechanism by which aPC reduces neutrophil migration is unknown, and no anti-inflammatory effects were found in this study [13].

Exogenous administration of aPC in animal models of endotoxemia and sepsis have shown an improvement of lung vascular injury, which is an important characteristic of sepsis [2,14,15]. aPC prevented lung edema, interstitial granulocyte infiltration and inhibited increase in TNF-α plasma levels. In the Prowess-study [1], a faster resolution of pulmonary dysfunction and increased ventilator-free days was observed [16]. The ARDS duration has not been assessed.

Another indication that the protein C system is disrupted in ALI/ARDS comes from the finding that lower protein C levels were found in the circulation and BAL fluid from patients with ALI and ARDS, which correlated with worse outcome, fewer ventilator-free days and more nonpulmonary organ failures [17].

Hence, our hypothesis is that systemic aPC will benefit patients with ALI/ARDS, as caused by inflammatory as well as infectious disorders, in terms of gas exchange, edema and capillary leak in these lungs, as well as in ventilator-days (duration of mechanical ventilation) or change in ventilatory mode.

## Primary objective and secondary objectives

We propose a placebo-controlled evaluation of the effect of aPC on pulmonary microvascular permeability (radio-isotope method [18] available at our institution), extravascular lung water, the lung injury score (LIS), gas exchange, radiographic abnormalities, duration of mechanical ventilation (if applied) or change in ventilatory mode in patients with ALI of inflammatory or infectious etiology. Patients who meet the PROWESS/NVIC inclusion criteria will not be included [1,19].

Secondly, we will assess several markers of coagulation and fibrinolysis in both the systemic circulation and the alveolar compartment using (mini) broncho-alveolar lavage (in the mechanically ventilated group).

Thirdly, we will assess markers of inflammation and apoptosis in the systemic circulation as well as in the alveolar compartment (when mechanically ventilated).

## Research design

# Patients

106 consecutive patients with ALI/ARDS

1. *Infectious* causes (evidence of pneumonia, SIRS + **single organ** respiratory failure with a PaO2/FiO2 ratio ≤ 300 (ALI) or ≤ 200 (ARDS)
2. *Inflammatory* causes (radiographic abnormalities on chest X-ray, SIRS + PaO2/FiO2 ratio ≤ 300)

**Study design**

Prospective, randomized, placebo-controlled, open-label study.

###### Setting

Two medical-surgical intensive care units and medium care units in two academic hospitals.

**Methods**

**Therapeutic protocol.**

Patients are treated with help of standard guidelines effective in our units. The full medical treatment will be under the discretion of the supervising staff-intensivists who are not directly involved in the study. When indicated, mechanical ventilation is performed after intratracheal intubation, in a pressure-controlled mode, aiming at a P max below 35 cm H2O at tidal volumes at or below 6 mL/kg predicted body weight. Prone position will be allowed. When indicated, noninvasive positive pressure ventilation will be performed using a standard face mask or helmet. Weaning from the ventilator and extubation will be strictly performed using a detailed local protocol.

Antibiotic therapy will be guided by Gram stains and cultures for pneumonia, according to our standardized approach.

Fluid therapy will consist of saline, gelatins or hydroxyethyl starches, in order to maintain arterial blood pressure (MAP > 70 mm Hg), diuresis (>30 ml/h) and EVLW (< 7 mL/kg).

**Study protocol.**

1. 53 patients with ALI/ARDS will receive aPC according to PROWESS protocol (24 μg/kg/hr during (in total) 96 hrs)[1], starting at inclusion, within a time-frame of 24 hours from diagnosis.
2. 53 patients with ALI/ARDS will receive placebo during 96 hrs.

## Standard aPC use

If the patient meets the criteria for aPC administration (severe sepsis + ≥ 2 failing organs, see reference 19) during the trial, the treating intensivist is permitted to initiate aPC treatment. Recent EMEA recommendations stated that “the use of aPC should be considered mainly in situations when therapy can be started **within 24 hours** after the onset of organ failure”. In addition, aPC should not be used in the acre of patients with single organ dysfunction, especially when they have had recent surgery (within 30 days). [unpublished data from ADDRESS and ENHANCE trials].

## Inclusion criteria.

- age 18 - 75 years
- weight < 135 kg
- Recent onset **ALI or ARDS** [30]
  - *Oxygenation*: P_a_O_2_/F_i_O_2_ ≤ 300 (**ALI) or**  ≤ 200 **(ARDS),** regardless of PEEP
  - *Chest radiograph*: uni – or bilateral infiltration seen on frontal chest radiograph
  - *Pulmonary capillary wedge pressure*: ≤18 mm Hg when measured or *no clinical evidence of left arterial hypertension* (lack of reaction to furosemide, no cardiac history, no dysfunction of echocardiography)
- **ALI** due to severe sepsis reflecting **single** organ failure
- **ARDS** due to severe sepsis reflecting **single** organ failure
- **ALI/ARDS** caused by inflammatory disorders of non-infectious origin
  - aspiration pneumonia
  - pulmonary contusion caused by trauma
  - vasculitis
  - acute pancreatitis
  - > 12 hrs post-surgery eg. thoracic or (cardio)vascular surgery
  - drug-induced lung injury eg. amiodarone, furandantin, etc.
  - bronchiolitis obliterans
  - massive transfusions
  - drug overdose
  - fat embolism
  - near drowning
  - reperfusion pulmonary edema
  - inhalation injury

**Exclusion criteria (according to PROWESS [1] and the NVIC guideline [19])**

Patients are not eligible when aPC is indicated: adult patients with severe sepsis with multiple organ failure when added to best standard care, mainly to be considered with 24 hours after the onset of multiple organ failure.

When NVIC inclusion criteria for aPC treatment are fulfilled [19].

1. absence of (seriously) limited life expectancy (non-sepsis related)
2. **all three** of the following:
3. ≥ 1 of the following known or suspected infection criteria:
   - - white cells in a normally sterile body fluid
     - perforated viscus
     - radiographic evidence of pneumonia in association with the production of purulent sputum
     - a syndrome associated with a high risk of infection (eg. ascending cholangitis)
4. ≥3 of modified SIRS criteria:
   - - a core temperature of ≥ 38^0^ C or ≤ 36^0^ C
     - a heart rate of ≥ 90 beats/min except in patients with a medical condition known to increase the heart rate or those receiving treatment that would prevent tachycardia
     - a respiratory rate ≥ 20 breaths/min or a PaCO_2_ ≤ 32 mm Hg or the use of mechanical ventilation for an acute respiratory process
     - a white cell count of ≥ 12000/mm^3^ or ≤ 4000/mm^3^ or a differential count showing > 10% immature neutrophils.
5. ≥ 2 of more sepsis-induced failing organs *or* shock:
   - - *Cardiovascular system dysfunction;* arterial systolic blood pressure is ≤ 90 mm Hg or the mean arterial pressure ≤ 70 mm Hg for at least 1 hour despite adequate fluid resuscitation, adequate intravascular volume status or the use of vasopressors in an attempt to maintain a systolic blood pressure of ≥ 90 mm Hg or a mean arterial pressure of ≥ 70 mm Hg.
     - *Kidney dysfunction;* urine output had to be <0.5 ml/kg of body weight/hr for 1 hour, despite adequate fluid resuscitation.
     - R*espiratory-system dysfunction*; the ratio of PaO2 to FiO2 had to be ≤ 250 (mm Hg) in the presence of other dysfunctional organs or systems or ≤ 200 (mm Hg) if the lung was the only dysfunctional organ.
     - *Hematologic dysfunction*; the platelet count had to be <80,000/mm3 or to have decreased by 50 percent in the 3 days preceding enrollment.
     - *Unexplained metabolic acidosis*; the pH had to be «7.30 or the base deficit had to be ≥ 5.0 mmol/liter in association with a plasma lactate level that was >1.5 times the upper limit of the normal value for the reporting laboratory (2.2 mmol/l)

**or** APACHE II score ≥ 25 at any moment during the current illness preceding the planned aPC administration.

**Further exclusion criteria according to Prowess [1].**

- - Thrombocytes lower than 30 * 10^9^/l
  - Any major surgery within 12 h before inclusion
  - Trauma patients at increased risk of bleeding
  - Acute bleeding
  - A history of severe head trauma that required hospitalization, intracranial surgery, or stroke within 3 months of study entry
  - Known intracranial abnormality such as aneurysms, tumor, arterio-venous malformation
  - Known hypercoagulability
    - Resistance to protein C
    - Hereditary deficiency of protein C, protein S, or antithrombin
    - Presence of anticardiolipin antibody, antiphospholipid antibody, lupus anticoagulant or homocystinemia
    - Recently documented (within 3 months of study entry) or highly suspected deep vein thrombosis or pulmonary embolism
  - A history of congenital bleeding diasthesis
  - Expected life expectancy less than 28 days (moribund state)
  - Preterminal illness
  - Pregnancy or breast feeding
  - Known portal hypertension with liver cirrhosis, esophageal varices or both
  - Epidural catheter
  - Body weight >135 kg
  - Chronic renal insufficiency
  - Participation in another clinical trial
  - Patients with immune system impairment
    - HIV-infected patients (CD4^+^ < 50/μl)
    - After bone-marrow, lung, liver, pancreas or small-bowel transplantation and treated with immunosuppressive therapy

## Concomitant medications

*Not allowed*

- Heparin in therapeutic doses (within 8 h of drug administration)
- LMWH in higher doses than as prophylaxis (within 12 h of study entry)
- Warfarin (within 7 days of study entry) **and** a PT exceeding the normal rane
- Aspirin higher than 650 mg (within 3 days of study entry)
- Thrombolytic therapy (within 3 days of study entry)
- GPIIb/IIIa inhibitors (within 7 days of study entry)
- Antithrombin-III at doses > 10.000 U (within 12 h of study entry)
- Protein C (within 24 h of study entry)
- Concomitant surfactant therapy or any other drug trial.

*Allowed*

- Steroids (up to 100 mg of hydrocortisone, 3 times daily)
- Selective decontamination of the gut with help of nonresorbable antibiotics (standard therapy in our institutes for patients with an expected duration of mechanical ventilation of at least 48 hrs)

**Procedure**

1. Informed consent [appendix I]
2. Clinical data (routine): demographic characteristics, APACHE II, comorbidity, site and type of infection, ventilator free days (both invasive and noninvasive), duration of ALI
3. Day 1-5, 7, 9, 11, 13, 15

Disease severity: SOFA, SAPS II, LIS [appendix II]

#### General hemodynamics

Specific hemodynamics using PiCCO technology (when indicated): extravascular lung water index (ELWI), pulmonary vascular permeability index (PVPI)

Respiratory: prone/supine, spontaneous/noninvasive/invasive, ventila-tion mode, FiO2, rate, peak-/plateau pressure, PEEP, PaO2/FiO2, PaO2/FiO2-versus-time curve, ABG, compliance, electrical impedance tomography (optional)

Laboratory:

Routine hematology/biochemistry: CRP, PCT

**Inflammatory mediators/biomarkers:** IL-1, Il-6, IL-10, MIF, TNFα, LBP, surfactant proteins (SP-A and SP-D), HII56, sE- selectin, BNP, HSP-70, VEGF

**markers of coagulation** **and fibrinolysis:** PC, aPC, PAI, TATc, PAA, TF, AP, t-PA, u-PA, soluble thrombomodulin

**markers** **of apoptosis:** nucleosomes, microparticles, NF-kB, sFasL

##### Chest X ray:

##### LIS [appendix II]

Scoring by radiologist who is blinded

1. Day 1, 3, 5, 7, 9, 11, 13, 15

mini-BAL (appendix III)

**infection markers**: leukocytes/neutrophils/culture

**inflammatory mediators/biomarkers:** IL-1, IL-6, IL-10, MIF, TNFα, sTREM-1, HTI156, sE-selectin, HSP-70, VEGF

**markers of coagulation and fibrinolysis**: PC, aPC, PAI, TATc, TF, AP, t-PA, soluble thrombomodulin

**markers of apoptosis**: ex vivo chemotaxis

1. Day 1 and 5

67gallium-transferrin pulmonary leak index (PLI) [see appendix IV]

CT thorax

Blinded radiologist

1. Day 1-4 during placebo and aPC infusion

Safety monitoring (appendix V: routine ICU monitoring, daily coagulation parameters and thrombocyte counts, (at least) daily physical examination with specific attention for bleeding.

Stopping rules will be applied according to Prowess [1]: the infusion will be interrupted 1 hour before any percutaneous procedure or major surgery and will be resumed 1 hour and 12 hours later, respectively.

**Primary and secondary measures**

**Primary:** 67gallium-transferrin pulmonary leak index (PLI)

Lung Injury Score

**Secondary:** extra-vascular lung water (PiCCO parameters)

gas exchange (compliance, PaO2/FiO2)

radiographic abnormalities (X-ray, CT)

change of ventilatory mode (noninvasive 🡪 invasive)

duration of mechanical ventilation

inflammatory mediators/biomarkers (blood, mini-BAL)

coagulation and fibrinolysis markers (blood, mini-BAL)

apoptosis markers

**DROP OUT**

If a patient is enrolled in this study, and subsequently develops severe sepsis with multiple organ failure, then it is the responsibility of the treating physician to determine the appropriate medical care to be provided to the patient based on the physician’s medical judgment. Such medical care might include the use of commercially available aPC (Xigris). If the medical care provided by the treating physician might be altered by knowing the patient’s treatment assignment, the investigator will request to be unblinded to the patient’s treatment assignment by calling the Medical Monitor. If this event occurs during the study infusion, the physician may stop the study drug infusion and begin another course of medical therapy as he or she deems appropriate. For example, if the attending physician would like to use aPC and the patient *is not* already receiving this drug, then the Medical Monitor will be contacted. If the patient is receiving the placebo, then the study drug will be stopped and the attending physician can then administer Xigris as he/she wishes. If the patient is already receiving aPC, then no change will be required. The primary data analysis will be carried out as intention to treat. For further information see statistical analysis plan.

**Statistics**

A sample size of 96 patients was calculated using a power calculation in which the anticipated difference of the PLI is 20% with an alpha of 0.05 and a power of 80% and a standard deviation of 40%. This power calculation is based upon the pooled data of all patients. Since we expect a drop out of 10% we will increase the sample size to n=106.

## The difference of the primary effect parameter (PLI) will be analyzed using the Mann-Whitney U-test. Groups will be compared with help of repeated measurements ANOVA, to evaluate the treatment effect on the pathophysiological and morbidity indictors (LIS).

## All randomized patients will be included in the primary analysis when post-baseline measurements are available, such as day 5 PLI measurements or day 1-5 Lung Injury scores.

It is anticipated that a small number of patients (<10%) will drop out of the study, primarily because of the development of severe sepsis and the desire of the primary treating physician to know if the patient is already being treated with APC. Statistically, these patients will be analyzed as intention to treat. Furthermore, a sensibility analysis per protocol will be performed also to understand the impact of drop outs on the trial results.

We will plan a sub-analysis after 30 patients in order to analyze the results of this study with exclusion of the patients who did not complete their placebo treatment because their physicians wish to give APC because of the development of severe sepsis. If the frequency of the development of severe sepsis is greater than anticipated, then the sample size of the trial will be increased by the number that would equal the number of patients that had been unblinded.

**References**

1. Bernard GR, et al. Efficacy and safety of recombinant human activated protein C for severe sepsis. N Engl J Med 2001;344:699-709.
2. Laterre PF, et al. Anticoagulant therapy in acute lung injury.
   Crit Care Med. 2003 Apr;31(4 Suppl):S329-36.
3. Matthay MA. Severe sepsis – a new treatment with both anticoagulant and antiinflammatory properties. N Engl J Med 2001;344;759-762.
4. Bernard GR. Drotrecogin alfa (activated) (recombinant human activated protein C) for the treatment of severe sepsis. Crit Care Med. 2003 Jan;31(1 Suppl):S85-93. Review.
5. Joyce DE, Grinell BW. Recombinant human activated protein C attenuates the inflammatory response in endothelium and monocytes by modulating nuclear factor-kappaB. Crit Care Med. 2002 May;30(5 Suppl):S288-93.
6. Abraham E. Coagulation abnormalities in acute lung injury and sepsis. Am J Respir Cell Mol Biol 2000;22:401-404.
7. Ware LB, Matthay MA. Medical Progress: the acute respiratory distress syndrome. N Eng J Med 2000;342:1334-1349.
8. Ware LB, Fang X, Matthay MA. Protein C and thrombomodulin in human acute lung injury. Am J Physiol Lung Cell Mol Physiol 2003;285:L514-L521.
9. Marshall JC. Inflammation, coagulopathy, and the pathogenesis of multiple organ dysfunction syndrome. Crit Care Med. 2001 Jul;29(7 Suppl):S99-106.
10. Schultz MJ, et al. Local activation and inhibition of fibrinolysis in the lung during ventilator-associated pneumonia. Thorax 2004;59:130-135.
11. Esmon CT. The protein C pathway. Chest 2003;124:26S-32S.
12. Hataji O, et al. Activation of protein C in the airways. Lung 2002:180;47-59.
13. Nick JA, et al. recombinant human aPC reduces endotoxin-induced pulmonary inflammation via inhibition of neutrophil chemotaxis. Blood, in press.
14. Murakami K, Okajima K, Uchiba M, Johno M, Nakagaki T, Okabe H, Takatsuki K. Activated protein C prevents LPS-induced pulmonary vascular injury by inhibiting cytokine production. Am J Physiol. 1997 Feb;272(2 Pt 1):L197-202.
15. Murakami K, Okajima K, Uchiba M, Johno M, Nakagaki T, Okabe H, Takatsuki K. Activated protein C attenuates endotoxin-induced pulmonary vascular injury by inhibiting activated leukocytes in rats. Blood. 1996 Jan 15;87(2):642-7.
16. Vincent JL, et al. Effects of drotrecogin alfa (activated) on organ dysfunction in the PROWESS trial. Crit Care Med 2003;31:834-840.
17. Matthay MA, Ware LB. Plasma protein C levels in patients with acute lung injury: prognostic significance. Crit Care Med 2004;32[suppl]:S229-S232.
18. Groeneveld ABJ. Radionuclide assessment of pulmonary microvascular permeability. Eur J Nucl Med 1997;24:449-460.
19. Girbes ARJ, et al. Toepassing drotrecogin alpha, geactiveerd proteine C (aPC) bij de behandeling van ernstige sepsis. NJCC 2003;10:285-288.
20. Groeneveld ABJ, Raijmakers PGHM. The ^67^Ga transferrin pulmonary leak index in patients at risk for the acute respiratory distress syndrome. Crit Care Med 1998;26:685-691
21. Raijmakers PGHM, Groeneveld ABJ, Schneider AJ, Teule GJJ, Lingen A van, Eysman L, Thijs LG. Transvascu­lar transport of ^67^Ga in the lungs after cardiopulmonary bypass surgery. Chest 1993;104:1825-1832.
22. Raijmakers PGHM, Groeneveld ABJ, Rauwerda JA, Schneider AJ, Teule GJJ, Hack CE, Thijs LG. Transient increase in interleukin-8 and pulmonary microvascular permea­bility following aortic surgery. Am J Respir Crit Care Med 1995;151:698-705.
23. Groeneveld ABJ. Radionuclide assessment of pulmonary microvascular permeability. Eur J Nucl Med 1997;24:449-461.
24. Raijmakers PGHM, Groeneveld ABJ, Rauwerda JA, Teule GJJ, Hack CE. Acute lung injury after aortic surge­ry: the relation between lung and leg microvas­cular permeabi­lity to ^111^Indium-transferrin and circulating mediators. Thorax 1997:52:866-871.
25. Groeneveld ABJ, Raijmakers PGHM, Teule GJJ, Thijs LG. The ^67^Gallium pulmonary leak index in assessing the seve­rity and course of the adult respiratory distress syndro­me. Crit Care Med. 1996;24:1467-1472.
26. Raijmakers PGHM, Groeneveld ABJ, Teule GJJ, Thijs LG. The diagnostic value of the ^67^Gallium pulmonary leak index in pulmonary edema. J Nucl Med 1996;37:1316-1322.
27. Groeneveld ABJ, Raijmakers PGHM. The ^67^Ga transferrin pulmonary leak index during pneumonia and associated adult respiratory distress syndrome. Clin Sci 1997;93:463-470.
28. McLuckie A. The COLD system of haemodynamic monitoring. Intens Care World 1996;13:24-28.
29. Godje O, Peyerl M, Seebauer T, Dewald O, Reichart B. Reproducibility of double indicator dilution measurements of in intrathoracic blood volume compartments, extravascular lung water, and liver function. Chest 1998;113:1070-1077.
30. Bernard GR, et al. The American-European Consensus Conference on ARDS. Definitions, mechanisms, relevant outcomes, and clinical trial coordination. Am J Respir Crit Care 1994;149:818-824.
31. Millo J, et al. Compartmentalisation of cytokines and cytokine inhibitors in ventilator-associated pneumonia. Intensive Care Med. 2004 Jan;30(1):68-74.

# Appendix I

Informed consent

Patients will only be included after written informed consent from the patient or the closest relative has been ob­tained and this will be recorded in the patients' records.

After recovery patients will be fully informed about the study protocol.

**Appendix II**

Lung Injury Score (LIS) according to Murray et al.

**Component Value Score**

1.chest X ray appearance no alveolar consolidation 0

alveolar consolidation confined to 1 quadrant 1

alveolar consolidation confined to 2 quadrants 2

alveolar consolidation confined to 3 quadrants 3

alveolar consolidation confined to 4 quadrants 4

2. hypoxaemia score > 40 0

(PaO2/FiO2) 30-40 1

23-29 2

13-22 3

< 13 4

3. PEEP score > 5 cm H_2_O 0

(when ventilated) 6-8 cm H_2_O 1

9-11 cm H_2_O 2

12-14 cm H_2_O 3

> 15 cm H_2_O 4

4. respiratory system > 80 ml/cm H_2_O 0

compliance score 60-79 ml/cm H_2_O 1

(when available) 40-59 ml/m H_2_O 2

20-39 ml/cm H_2_O 3

< 19 ml/cm H_2_O 4

(Am Rev Respir Dis 1988; 138:720-723)

**Appendix III.**

We will use a non-directed bronchial lavage technique by installing 20 ml of sterile 0.9% saline via a standard 50 cm, 14 gauge tracheal suction catheter as described elsewhere [10,31]. In short, the distal end of the catheter was introduced via the endotracheal tube and advanced until significant resistance was encountered. Immediately, after installation over 10-15 seconds, fluid was aspirated before withdrawal of the catheter. Generally, 5-10 ml fluid will be recovered [10].

**Appendix IV.**

## Measurements of PLI

For the non-invasive assessment of pulmonary microvascular permeabi­li­ty a new technique developed by our group will be used, the ^67^Ga-transferrin pulmonary leak index PLI, involving ^67^ Ga bound to circulating transferrin in vivo, and ^99m^Tc in bound to erythrocytes in vitro [18, 20-27]. The technique been described elsewhere. In short, 10 min after intravenous administration of 5 mg pyrophosphate (TechneScan, Mallinckrodt Medical, Petten), 10 mL of blood is obtained and equilibrated with 300 μCi (11 MBq) ^99m^Tc. Ten min later the blood is reinjected. In vivo labeling of transferrin occurs after injection of ^67^Ga-citrate (100 μCi, 4 MBq for the preinfusion study and 100 μCi, 4 MBq, for the postinfusion study). This is the only currently available non-invasive technique to measure pulmonary microvascular permea­bility and is available at only few places in the world. For the measure­ment of EVLW the thermal/dye thermodilution technique with help with the so called COLD^R^ machine (Pulsion, Munchen, Germany) will be used, yielding the gold standard for assessment of pulmonary edema [20,28,29]. This measurement involves central venous injection of a dye and thermal bolus and concomitant registration of the dye dilution and thermal shift in the femoral artery, with help of a 5F catheter for pressure monitoring, equipped with a thermistor and fiberoptic. All other (hemodynamic and labora­tory) variables are stand­ard.

**Patient inconvenience.**

Apart from blood sampling, the radioactivity dose of ^99m^Tc (physical half live 6 hr) and of ^67^Ga (physical half live 78 h) used for the study is routine for measurement of the PLI (9) and is safe. The absorbed dose is 444 μSv for ^67^Ga and 94 μSv for ^99m^Tc per study, at a natural background of about 2400 μSv per year. Hence, the additional radiation dose is about 40 % of the yearly load, which is acceptable. Since inclusion criteria include an arterial (5F fiberoptic, femoral artery) in place, the thermal dye dilution technique can be used to evaluate the EVLW without any additional discomfort for the patients. The additional blood sampling for the study will not exceed 100 mL.

**Ethical considerations and informed consent.**

After protocol approval by the sponsor, the proposed study in this program will submitted to the scientific and ethical committees of the Institute of Cardiovascular Research at the VU (ICARVU) and the VU University Medical Center, respectively, for approval. The study will be performed in accordance with the Declaration of Helsinki (see Appendix VI). The Hospital Committee on Ethics will have to approve the protocol. Patients will only be included after informed consent from the patient or the closest relative has been ob­tained and this will be recorded in the patients' records. The sponsor will have free access to the (anony­mous) patient data, if requested.

**The research group.**

The Department of Intensive Care is, as far as research is concerned, part of the Institute of Cardiovascular Research at the Vrije Universiteit (ICARVU), forming together with a similar institution at the University of Maastricht, the research school CARMA - Cardiovascular Research school Maastricht-Amsterdam. The group has a large expe­rience with this type of studies and the methods mentioned. The studies are to be performed in close collaboration with the staff of the Intensive Care and the Department of Nuclear Medicine of our hospi­tal. The organization and analysis of the studies, however, are in the hands of the principal investigator, helped by advisors that partici­pate in the study. We closely collaborate with the Department of Intensive Care of the Academic Medical Center in Amsterdam, a very active research group with a special focus on pneumonia, lung inflammation and coagulation.

**Appendix** **V.**

Investigators are responsible for the safety of subjects who entered this study and for appropriate medical care of study participants during the study in connection with protocol procedures.

Adverse events

**All** adverse events occurring after enrollment will be reported to Lilly or designee by entry on a CRF page titled ‘preexisting conditions and study adverse events’.

Any **serious** AE (SEA) will be reported to Lilly or designee immediately, by telephone, except when the event is experienced prior to assignment to treatment and the investigator does not feel that the vent may have been caused by a protocol procedure. All SEA will also be reported by Fax.

Dosage adjustments are not allowed. If a patient’s treatment is discontinued as a result of an AE , the circumstances and data leading to this discontinuation, will be clearly documented, using designated CRFs. For any untoward event(s), the patient will be followed until the vent resolves or is explained, with frequency of follow-up at the discretion of the investigator.

SEA

- prolonged inpatient hospitalization or rehospitalization
- a life-threatening experience (ie, immediate risk of dying)
- cancer
- severe or permanent disability
- congenital anomaly
- meets criteria for a serious bleeding event (see next section)

Bleeding events

- adrenal hemorrhage
- severe anemia
- bloody diarrhea
- cerebral hemorrhage
- duodenal ulcer hemorrhage
- ecchymosis
- esophageal hemorrhage
- gastrointestinal hemorrhage
- hematemesis
- hematochezia
- hemolysis
- hemopercardium
- hemoperitoneum
- hemoptysis
- hemorrhage
- hemorrhage of colon
- hemorrhage of ileum
- hemorrhage of jejunum
- hemorrhagic colitis
- hemorrhagic cystitis
- hemorrhagic enterocolitis
- hemorrhagic gastritis
- hemorrhagic pancreatitis
- hemorrhagic proctitis
- hemothorax
- intracranial hemorrhage
- lung hemorrhage

Serious bleeding events

These are defined as bleeding events that meet the criteria for reporting as SEA or are associated with any of the following outcomes or treatment:

- - 1. death
    2. intracranial hemorrhage
    3. require treatment by transfusion therapy equal or greater than 3 units RBC/day for 2 consecutive days

all serious bleeding events will be reported as SEA.

If a bleeding event meeting the above criteria occurs during aPC infusion, the infusion will be discontinued and a SAE CRF should be completed.

SEAs will be collected starting from the time the patients receives aPC and up to 28 days thereafter.

Investigators will determine relatedness of an event to aPC based on a temporal relationship to aPC infusion as well as if the event is anticipated or unexplained given the patient’s clinical course, previous medical conditions, and concomitant medications. An event should be recorded as ‘drug-related’ if the investigator believes it is reasonably related to aPC.

Study-specific clinical outcomes of ALI/ARDS are exempt from all AE and EA, unless the investigator deems them related to the use of aPC.

SEA occurring after a subject is discontinued from the study will **not** be reported unless the investigator feels that the event may have been caused by aPC or a protocol procedure.

Mortality reporting

All deaths occurring within 28 days from initiation of aPC or placebo will be reported in the CRF.

**Appendix VI.**

**WORLD MEDICAL ASSOCIATION DECLARATION OF HELSINKI**

**Ethical Principles for Medical Research Involving Human Subjects**

Adopted by the 18th WMA General Assembly, Helsinki, Finland, June 1964, and amended by the 29th WMA General Assembly, Tokyo, Japan, October 1975; 35th WMA General Assembly, Venice, Italy, October 1983; 41st WMA General Assembly, Hong Kong, September 1989; 48th WMA General Assembly, Somerset West, Republic of South Africa, October 1996 and the 52nd WMA General Assembly, Edinburgh, Scotland, October 2000.

Note of Clarification on Paragraph 29 added by the WMA General Assembly, Washington 2002

Note of Clarification on Paragraph 30 added by the WMA General Assembly, Tokyo 2004

A. INTRODUCTION

1. The World Medical Association has developed the Declaration of Helsinki as a statement of ethical principles to provide guidance to physicians and other participants in medical research involving human subjects. Medical research involving human subjects includes research on identifiable human material or identifiable data.

2. It is the duty of the physician to promote and safeguard the health of the people. The physician's knowledge and conscience are dedicated to the fulfillment of this duty.

3. The Declaration of Geneva of the World Medical Association binds the physician with the words, "The health of my patient will be my first consideration," and the International Code of Medical Ethics declares that, "A physician shall act only in the patient's interest when providing medical care which might have the effect of weakening the physical and mental condition of the patient."

4. Medical progress is based on research which ultimately must rest in part on experimentation involving human subjects.

5. In medical research on human subjects, considerations related to the well-being of the human subject should take precedence over the interests of science and society.

6. The primary purpose of medical research involving human subjects is to improve prophylactic, diagnostic and therapeutic procedures and the understanding of the etiology and pathogenesis of disease. Even the best proven prophylactic, diagnostic, and therapeutic methods must continuously be challenged through research for their effectiveness, efficiency, accessibility and quality.

7. In current medical practice and in medical research, most prophylactic, diagnostic and therapeutic procedures involve risks and burdens.

8. Medical research is subject to ethical standards that promote respect for all human beings and protect their health and rights. Some research populations are vulnerable and need special protection. The particular needs of the economically and medically disadvantaged must be recognized. Special attention is also required for those who cannot give or refuse consent for themselves, for those who may be subject to giving consent under duress, for those who will not benefit personally from the research and for those for whom the research is combined with care.

9. Research Investigators should be aware of the ethical, legal and regulatory requirements for research on human subjects in their own countries as well as applicable international requirements. No national ethical, legal or regulatory requirement should be allowed to reduce or eliminate any of the protections for human subjects set forth in this Declaration.

B. BASIC PRINCIPLES FOR ALL MEDICAL RESEARCH

10. It is the duty of the physician in medical research to protect the life, health, privacy, and dignity of the human subject.

11. Medical research involving human subjects must conform to generally accepted scientific principles, be based on a thorough knowledge of the scientific literature, other relevant sources of information, and on adequate laboratory and, where appropriate, animal experimentation.

12. Appropriate caution must be exercised in the conduct of research which may affect the environment, and the welfare of animals used for research must be respected.

13. The design and performance of each experimental procedure involving human subjects should be clearly formulated in an experimental protocol. This protocol should be submitted for consideration, comment, guidance, and where appropriate, approval to a specially appointed ethical review committee, which must be independent of the investigator, the sponsor or any other kind of undue influence. This independent committee should be in conformity with the laws and regulations of the country in which the research experiment is performed.

The committee has the right to monitor ongoing trials. The researcher has the obligation to provide monitoring information to the committee, especially any serious adverse events. The researcher should also submit to the committee, for review, information regarding funding, sponsors, institutional affiliations, other potential conflicts of interest and incentives for subjects.

14. The research protocol should always contain a statement of the ethical considerations involved and should indicate that there is compliance with the principles enunciated in this Declaration.

15. Medical research involving human subjects should be conducted only by scientifically qualified persons and under the supervision of a clinically competent medical person. The responsibility for the human subject must always rest with a medically qualified person and never rest on the subject of the research, even though the subject has given consent.

16. Every medical research project involving human subjects should be preceded by careful assessment of predictable risks and burdens in comparison with foreseeable benefits to the subject or to others. This does not preclude the participation of healthy volunteers in medical research. The design of all studies should be publicly available.

17. Physicians should abstain from engaging in research projects involving human subjects unless they are confident that the risks involved have been adequately assessed and can be satisfactorily managed. Physicians should cease any investigation if the risks are found to outweigh the potential benefits or if there is conclusive proof of positive and beneficial results.

18. Medical research involving human subjects should only be conducted if the importance of the objective outweighs the inherent risks and burdens to the subject. This is especially important when the human subjects are healthy volunteers.

19. Medical research is only justified if there is a reasonable likelihood that the populations in which the research is carried out stand to benefit from the results of the research.

20. The subjects must be volunteers and informed participants in the research project.

21. The right of research subjects to safeguard their integrity must always be respected. Every precaution should be taken to respect the privacy of the subject, the confidentiality of the patient's information and to minimize the impact of the study on the subject's physical and mental integrity and on the personality of the subject.

22. In any research on human beings, each potential subject must be adequately informed of the aims, methods, sources of funding, any possible conflicts of interest, institutional affiliations of the researcher, the anticipated benefits and potential risks of the study and the discomfort it may entail. The subject should be informed of the right to abstain from participation in the study or to withdraw consent to participate at any time without reprisal. After ensuring that the subject has understood the information, the physician should then obtain the subject's freely-given informed consent, preferably in writing. If the consent cannot be obtained in writing, the non-written consent must be formally documented and witnessed.

23. When obtaining informed consent for the research project the physician should be particularly cautious if the subject is in a dependent relationship with the physician or may consent under duress. In that case the informed consent should be obtained by a well-informed physician who is not engaged in the investigation and who is completely independent of this relationship.

24. For a research subject who is legally incompetent, physically or mentally incapable of giving consent or is a legally incompetent minor, the investigator must obtain informed consent from the legally authorized representative in accordance with applicable law. These groups should not be included in research unless the research is necessary to promote the health of the population represented and this research cannot instead be performed on legally competent persons.

25. When a subject deemed legally incompetent, such as a minor child, is able to give assent to decisions about participation in research, the investigator must obtain that assent in addition to the consent of the legally authorized representative.

26. Research on individuals from whom it is not possible to obtain consent, including proxy or advance consent, should be done only if the physical/mental condition that prevents obtaining informed consent is a necessary characteristic of the research population. The specific reasons for involving research subjects with a condition that renders them unable to give informed consent should be stated in the experimental protocol for consideration and approval of the review committee. The protocol should state that consent to remain in the research should be obtained as soon as possible from the individual or a legally authorized surrogate.

27. Both authors and publishers have ethical obligations. In publication of the results of research, the investigators are obliged to preserve the accuracy of the results. Negative as well as positive results should be published or otherwise publicly available. Sources of funding, institutional affiliations and any possible conflicts of interest should be declared in the publication. Reports of experimentation not in accordance with the principles laid down in this Declaration should not be accepted for publication.

C. ADDITIONAL PRINCIPLES FOR MEDICAL RESEARCH COMBINED WITH MEDICAL CARE

28. The physician may combine medical research with medical care, only to the extent that the research is justified by its potential prophylactic, diagnostic or therapeutic value. When medical research is combined with medical care, additional standards apply to protect the patients who are research subjects.

29. The benefits, risks, burdens and effectiveness of a new method should be tested against those of the best current prophylactic, diagnostic, and therapeutic methods. This does not exclude the use of placebo, or no treatment, in studies where no proven prophylactic, diagnostic or therapeutic method exists (see footnote 1).

30. At the conclusion of the study, every patient entered into the study should be assured of access to the best proven prophylactic, diagnostic and therapeutic methods identified by the study (see footnote 2).

31. The physician should fully inform the patient which aspects of the care are related to the research. The refusal of a patient to participate in a study must never interfere with the patient-physician relationship.

32. In the treatment of a patient, where proven prophylactic, diagnostic and therapeutic methods do not exist or have been ineffective, the physician, with informed consent from the patient, must be free to use unproven or new prophylactic, diagnostic and therapeutic measures, if in the physician's judgment it offers hope of saving life, re-establishing health or alleviating suffering. Where possible, these measures should be made the object of research, designed to evaluate their safety and efficacy. In all cases, new information should be recorded and, where appropriate, published. The other relevant guidelines of this Declaration should be followed.

1 **Note of clarification on paragraph 29 of the WMA Declaration of Helsinki**

The WMA hereby reaffirms its position that extreme care must be taken in making use of a placebo-controlled trial and that in general this methodology should only be used in the absence of existing proven therapy. However, a placebo-controlled trial may be ethically acceptable, even if proven therapy is available, under the following circumstances:

- Where for compelling and scientifically sound methodological reasons its use is necessary to determine the efficacy or safety of a prophylactic, diagnostic or therapeutic method; or

- Where a prophylactic, diagnostic or therapeutic method is being investigated for a minor condition and the patients who receive placebo will not be subject to any additional risk of serious or irreversible harm.

All other provisions of the Declaration of Helsinki must be adhered to, especially the need for appropriate ethical and scientific review.

2 **Note of clarification on paragraph 30 of the WMA Declaration of Helsinki**

The WMA hereby reaffirms its position that it is necessary during the study planning process to identify post-trial access by study participants to prophylactic, diagnostic and therapeutic procedures identified as beneficial in the study or access to other appropriate care. Post-trial access arrangements or other care must be described in the study protocol so the ethical review committee may consider such arrangements during its review.

9.10.2004

**Appendix VII.**

Verzekeringsparagraaf.

Ingevolge art. 7 van de Wet medisch -wetenschappelijk onderzoek met mensen (Staatsblad 1998, 161) is door de verrichter van het onderzoek, het VUmc, een verzekering afgesloten die de door het onderzoek veroorzaakte schade door dood of letsel van de deelnemende proefpersonen dekt. Deze verzekering is afgesloten bij Onderlinge Waarborgmaatschappij Centramed b.a., Postbus 90504, 2509 LM ‘s Gravenhage. De verzekering voldoet aan de bepalingen van het Besluit verplichte verzekering bij medisch-wetenschappelijk onderzoek met mensen (Stbl. 2003, 266). Aan het onderzoek deelnemende proefpersonen zullen schriftelijk worden geïnformeerd over de verzekering.

**Appendix VIII.**

De onafhankelijk arts voor de INFALI-studie is Dr. A.A. van Bodegraven, algemeen telefoonummer 020-4444444, vragen naar sein 589.
